# Comparative genomics reveals that a fish pathogenic bacterium *Edwardsiella tarda* has acquired the locus of enterocyte effacement (LEE) through horizontal gene transfer

**DOI:** 10.1186/1471-2164-14-642

**Published:** 2013-09-22

**Authors:** Yoji Nakamura, Tomokazu Takano, Motoshige Yasuike, Takamitsu Sakai, Tomomasa Matsuyama, Motohiko Sano

**Affiliations:** 1 National Research Institute of Fisheries Science, Fisheries Research Agency, 2-12-4 Fukuura, Kanazawa, Yokohama 236-8648, Japan; 2 National Research Institute of Aquaculture, Fisheries Research Agency, 422-1 Nakatsuhamaura, Minami-ise, Mie 516-0193, Japan; 3 Tamaki Laboratory, National Research Institute of Aquaculture, Fisheries Research Agency, Tamaki, Mie 519-0423, Japan; 4 Current address: Department of Marine Biosciences, Tokyo University of Marine Science and Technology, Minato-ku, Konan 4-5-7, Tokyo 108-8477, Japan

## Abstract

**Background:**

*Edwardsiella tarda* is an enterobacterium which causes edwardsiellosis, a fatal disease of cultured fishes such as red sea bream, eel, and flounder. Preventing the occurrence of *E. tarda* infection has thus been an important issue in aquaculture. *E. tarda* has been isolated from other animals and from many environments; however, the relationship between the genotype and evolutionary process of this pathogen is not fully understood. To clarify this relationship, we sequenced and compared the genomes of pathogenic and non-pathogenic *E. tarda* strains isolated from fish, human, and eel pond using next-generation sequencing technology.

**Results:**

Eight strains of *E. tarda* were sequenced with high accuracy (>99.9%) with coverages from 50- to 400-fold. The obtained reads were mapped to a public reference genome. By comparing single nucleotide and insertion/deletion polymorphisms, we found that an attenuated strain of *E. tarda* had a loss-of-function mutation in a gene related to the type III secretion system (T3SS), suggesting that this gene is involved in the virulence of *E. tarda*. A comprehensive gene comparison indicated that fish pathogenic strains possessed a type VI secretion system (T6SS) and pilus assembly genes in addition to the T3SS. Moreover, we found that an *E. tarda* strain isolated from red sea bream harbored two pathogenicity islands of T3SS and T6SS, which were absent in other strains. In particular, this T3SS was homologous to the locus of enterocyte effacement (LEE) in enteropathogenic and enterohemorrhagic *Escherichia coli*. Evolutionary analysis suggested that this locus, here named Et-LEE (*E. tarda* LEE), was introgressed into the *E. tarda* genome through horizontal transfer.

**Conclusions:**

We found significant differences in the presence/absence of virulence-related genes among *E. tarda* strains, reflecting their evolutionary relationship. In particular, a single genotype previously proposed for fish-pathogenic strains may be further divided into two subgroups. Furthermore, the current study demonstrated, for the first time, that a fish pathogenic bacterium carried a LEE-like pathogenicity island which was previously reported only in zoonotic pathogenic enterobacteria. These findings will contribute to the exploration of strain-specific drug targets against *E. tarda* in aquafarms, while also shedding light on the evolution of pathogenesis in enterobacteria.

## Background

*Edwardsiella tarda*, a member of the family Enterobacteriaceae, has been isolated from a variety of animals including fish and mammals [1]. In pathology, this bacterium is a known causative agent of a fish disease (e.g. gangrene and septicemia) named edwardsiellosis. Ever since the first report of edwardsiellosis in 1959 [1], the mass mortality of fish caused by this bacterium has been a serious issue in aquaculture [2]. *E. tarda* can infect a variety of fish species, including Japanese eel (*Anguilla japonica*), European eel (*Anguilla anguilla*), Japanese flounder (*Paralichthys olivaceus*), turbot (*Scophthalmus maximus*), yellowtail (*Seriola quinqueradiata*), red sea bream (*Pagrus major*), channel catfish (*Ictalurus punctatus*), and tilapia (*Oreochromis mossambicus*). *E. tarda* also causes diarrhea in humans (reviewed in [3-5]).

The type and virulence of the *E. tarda* strains have been examined by serological analysis and infection test, respectively. The isolates from Japanese eel, Japanese flounder and eel pond, were classified into four serotypes (A, B, C, and D) by the O-agglutination test [6,7]. The *E. tarda* that are highly virulent to fish are serotype A strains, but these strains do not always share the same biological traits. In particular, atypical serotype A strains of *E. tarda* isolated from red sea bream and yellowtail were non-motile, unlike the more typical serotype A strains [8]. To investigate the virulence of *E. tarda* in fish, the infection test was performed using both the Japanese flounder and red sea bream as hosts. While all the serotype A strains of *E. tarda* are, in principle, virulent to Japanese flounder, the atypical strains were reported to be virulent only in red sea bream [9].

Regarding the genomic data of *E. tarda*, a complete genome sequence of the turbot pathogenic strain EIB202, was reported in 2009 [10] and strain FL6-60 was sequenced in 2011 [11]. The genome sequence of the human pathogenic strain ATCC23685 was also determined and annotated, but the sequence is still fragmented. In addition, the complete genome sequence of *Edwardsiella ictaluri*[12], a close relative of *E. tarda* and causative agent of enteric septicemia in catfish, is currently available [13]. A recent whole genome comparison of multiple *E. tarda* strains showed that *E. tarda* genotypes were broadly clustered into two groups, EdwGI and EdwGII, which consisted of strains that were isolated mainly from fish and human, respectively [14]. EdwGI represents a genotype of fish pathogens in the *Edwardsiella* lineage and the genes of virulence factors such as type III secretion system (T3SS), type VI secretion system (T6SS), hemolysin, flagellin, adhesin, invasin, and fimbriae have been identified in strains from this group [2,14].

The relationships between the EdwGI and EdwGII genotypes and the A–D serotypes are not fully understood. Serotype A strains are virulent to fish, indicating that these strains are evolutionarily closely related to the EdwGI genotype. On the other hand, two unique DNA sequences from atypical serotype A strains have been detected. These DNA sequences were found to encode a novel T6SS and the type V secretion system (T5SS) [15]. Thus, there is a possibility that the virulence mechanism of serotype A/EwdGI *E. tarda* may differ between the typical and atypical strains, consistent with the reported host specificity in the infection test. In this study, we sequenced the genomes of four serotype *E. tarda* isolates (serotypes A-D) from aquaculture fishes or environmental water, and performed comparative analyses of the structure of the genomes and their virulence-related gene repertoire using the reference genome sequences such as those of EIB202 and ATCC23685. We demonstrated that fish-pathogenic and environmental *E. tarda* were clearly distinguishable at the sequence and gene repertoire level, and found that a single genotype proposed previously for fish-pathogenic strains could be further classified into two genotypes, typical and atypical. Strikingly, we report that an atypical strain of *E. tarda* has a pathogenicity island that is homologous to the pathogenicity islands of virulent *Escherichia coli* strains, which are causative agents of outbreaks of human foodborne illness.

## Methods

### Strains

For genome sequencing, we selected eight strains of *E. tarda* (Table 1), seven of which were of the four major serotypes A to D. Serotype A strains NUF806, E22 and FPC503 were isolated from Japanese flounder, Japanese eel, and red sea bream, respectively. The E22 strain has undergone attenuation during cultivation, and FPC503 is a non-motile atypical strain. NUF806 was kindly donated by Prof. Kanai (Nagasaki University, Japan). The SU100 (serotype C), SU138 (serotype B), SU244 (serotype D), and SU117 (undetermined) strains are environmental (non-pathogens): SU138 was isolated from the gut of a healthy eel, and the other three were from eel ponds. We also sequenced the genome of a publicly available strain, ATCC23685, which is a causative agent of human diarrhea, and used the data to evaluate the sequence accuracy in this study.

**Table 1:** ***E. tarda *** **strains sequenced in this study**

	**Strain**	**Source**	**Characteristics**	**Place of isolation**	**Isolation year**
Fish-pathogenic	NUF806	flounder (kidney)	serotype A	Nagasaki, Japan	1997
	E22	eel (blood)	serotype A attenuated during cultivation	Shizuoka, Japan	1972
	FPC503	red sea bream (kidney)	serotype A nonmotile	Nagasaki, Japan	1980
Non-pathogenic	SU100	eel pond	serotype C	Shizuoka, Japan	1980
	SU117	eel pond	N.D.	Shizuoka, Japan	1980
	SU138	eel gut	serotype B	Shizuoka, Japan	1980
	SU244	eel pond	serotype D	Shizuoka, Japan	1988
Reference strain	ATCC23685	human	O1958: H18	USA	N.D.

To compare the genome sequences of the eight *E. tarda* strains with the genomes of related species, we downloaded the sequences of three *E. tarda* strains, EIB202 [GenBank:NC_013508] and its plasmid pEIB202 [GenBank:NC_013509], FL6-60 [GenBank:CP002154] and plasmid pFL6-60 [GenBank:CP002155], and ATCC23685 [GenBank:ADGK01000000], as well as the complete genome sequence of the *E. ictaluri* strain 93–146 [GenBank:NC_012779]. For phylogenetic analysis, the nucleotide sequences of DNA gyrase subunit B genes (*gyrB*) were extracted from the genome data and from the unannotated contig data of *E. tarda* 080813 [GenBank:AFJH01000000] and *E. ictaluri* ATCC33202 [GenBank:AFJI01000000]. To compare the synteny of the pathogenicity islands in *E. tarda*, enteropathogenic *Escherichia coli* O127 [GenBank:FM180568], enteropathogenic *E. coli* O157 [GenBank:NC_002655], and *Pantoea ananatis* LMG 20103 [GenBank:NC_013956] were also downloaded from the GenBank database.

### Sample preparation and genome sequencing

The eight strains of *E. tarda* were individually cultured in 10 ml of heart infusion broth at 25°C for 20 hours. Bacterial cells were collected by centrifugation for 10 min at 8000 × g. Genomic DNA of each strain was extracted from the bacterial pellets using Maxwell 16 DNA Purification Kit (Promega Corporation, Madison, WI). Paired-end shotgun libraries (insert sizes of 300–400 bp) were prepared from 1–3 μg of genomic DNA using Paired-End DNA Sample Prep kit and Multiplexing Sample Preparation Oligonucleotide kit (Illumina Inc., San Diego, CA) according to the manufacturer’s protocols. The DNA concentration of each library was analyzed on a high sensitivity DNA chip with an Agilent 2100 bioanalyzer (Agilent Technologies, Palo Alto, CA). Each library (7–11 pM) was subjected to cluster amplification on a Paired End Flow Cell v4 with a cBot instrument and then sequenced on an Illumina Genome Analyzer IIx for 2 × 76 cycles using Illumina Sequencing kit v4 reagent (Illumina Inc.).

Additional whole genome shotgun sequencing of *E. tarda* strain FPC503 was performed using Roche 454 GS-FLX+ Titanium sequencing platform. Using the Covaris instrument (Covaris Inc., Woburn, MA), 1 μg of the genomic DNA was sheared into 1,500-bp fragments. A 454-pyrosequencing library was constructed from the sheared DNA by GS Titanium Rapid Library Preparation Kit (Roche Diagnostics, Branford, CT). Pyrosequencing was performed using 1/4 region of a 70 mm × 75 mm Titanium PicoTiter plate according to the manufacturer’s protocols (Roche Diagnostics). The short read sequence data that we obtained have been deposited in DDBJ/EMBL/GenBank [DDBJ: DRA001012].

### SNP detection and *de novo* assembly

We directly mapped the short reads obtained from the Illumina Genome Analyzer IIx to the genome sequence of strain EIB202, and detected single nucleotide polymorphisms (SNPs) and insertions/deletions (INDELs) using the program package, CASAVA (Illumina Inc.). *De novo* assembly was carried out using the ABySS program [16] with its parameter optimized manually. Briefly, we parameterized the k-mers based on a self-BLASTN search result between the assembled contigs. Overproduced contigs often contain redundant DNA regions which are similar to each other at the sequence level; these regions are considered to be caused by misassembly. Thus, we optimized k-mers in which the contig N50 increased and the redundant regions decreased (Additional file 1 Figures S1 and Additional file 2: and Figure S2). The 454 reads of *E. tarda* strain FPC503 were assembled into contigs with Newbler ver. 2.8 (Roche Diagnostics). The nucleotide sequences which we obtained were corrected by mapping the Illumina reads onto the contigs using BWA software [17]. The complete T6SS locus of FPS503 was constructed by joining two contigs using a genome walking method (BEX Co., Ltd., Tokyo, Japan).

### Gene prediction, annotation, and horizontal gene transfer

Open reading frames (ORFs) in each assembled genome sequence were predicted by a combination of two gene-finding programs, Glimmer3 [18] and GeneMarkS [19]. ORFs predicted by either of these programs were considered as potential protein-encoding genes. Gene function was inferred by BLASTP [20] searches against the NCBI *nr* database (as of 6th December, 2012) with an E-value <10^-5^ cutoff. An all-versus-all BLASTP search was performed among the genes in 10 strains of *E. tarda* (NUF806, E22, FPC503, SU100, SU117, SU138, SU244, and three public strains, EIB202, FL6-60 and ATCC23685), and an *E. ictaluri* strain 93–146 with an E-value <10^-10^ cutoff. An orthologous gene pair was defined as one reciprocal best hit. The ‘core’ gene that was conserved among the eleven strains was defined as the gene set in which any pair was defined as orthologous. The presence or absence of genes among the strains was tabularized as a matrix, and hierarchically clustered by an a function in *R* package, *hclust*. The absence of genes was confirmed by BLASTN with an E-value <10^-3^ cutoff against the assembled genome sequences. The horizontally transferred genes from other species were inferred using a Markov model method [21] which computed a horizontal transfer index (HT index) for each gene from the training model of coding and non-coding nucleotide compositions in the *E. tarda* genome. Genes with significantly low HT indices (*p* <0.005) were considered as genes that were recently transferred from different species.

### Molecular phylogenetic analyses

For molecular phylogenetic analysis, multiple sequence alignments were constructed by the MAFFT program [22]. Each alignment was first calculated using the deduced amino acid sequences, and then reversely translated to the nucleotide sequences. Evolutionary distances between the nucleotide sequences were calculated by Kimura’s two parameter method [23]. The phylogenetic trees were constructed by the neighbor-joining method [24] using MEGA5 [25].

## Results and discussion

### Genome assembly

The complete genome sequences of the eight *E. tarda* strains, E22, NUF806, FPC503, SU100, SU117, SU138, SU244, and ATCC23685, ranged in length from 3.63 to 3.96 Mb (Table 2). The estimated genome sizes were similar to those of the previously determined strains (EIB202: 3,760,463 bp; FL6-60: 3,684,607 bp) and *E. ictaluri* (93–146: 3,812,315 bp). The GC content ranged from 57.2% to 59.8%. The GC content of the three fish-pathogenic strains (NUF806, E22 and FPC503) was close to that of EIB202 (59.7%) and FL6-60 (59.8%) and around 2% higher than the GC content of the other four strains (SU100, SU117, SU138, and SU244). The four strains with the lower GC content are the environmental strains that were isolated either from pond or healthy eel gut, and their GC content was similar to that of *E. ictaluri* (57.44%). Thus we found that the fish-pathogenic and environmental strains of *E. tarda* were distinct from each other at the GC level.

**Table 2:** **Assembly and gene statistics of *E. tarda *** **genomes**

**Strain**	**Contig**	**Total size (bp)**	**Mean contig size (bp)**	**Longest contig size (bp)**	**N50 (bp)**	**GC%**	**Protein-coding genes**	**Known genes**
This study								
NUF806	59	3,751,597	63,586	339,184	257,179	59.77	3,590	3,517
E22	77	3,962,523	51,461	361,787	254,731	59.35	3,868	3,759
FPC503	97	3,952,758	40,750	277,956	191,777	59.11	3,882	3,562
SU100	71	3,628,706	51,108	682,159	340,928	57.26	3,404	3,277
SU117	98	3,632,832	37,069	372,253	222,307	57.33	3,425	3,258
SU138	172	3,761,148	21,867	527,925	232,606	57.32	3,499	3,337
SU244	134	3,745,746	27,953	665,778	222,315	57.18	3,528	3,357
ATCC23685	123	3,655,430	29,718	608,143	256,355	57.24	3,434	3,343
Public data								
EIB202	1	3,760,463				59.73	3,588	
FL6-60	1	3,684,607				59.81	3,256	
ATCC23685	87	3,744,568	43,041	2,378,503		57.16	3,964	
*E. ictaluri* 93-146	1	3,812,315				57.44	3,784	

**Figure 1:**
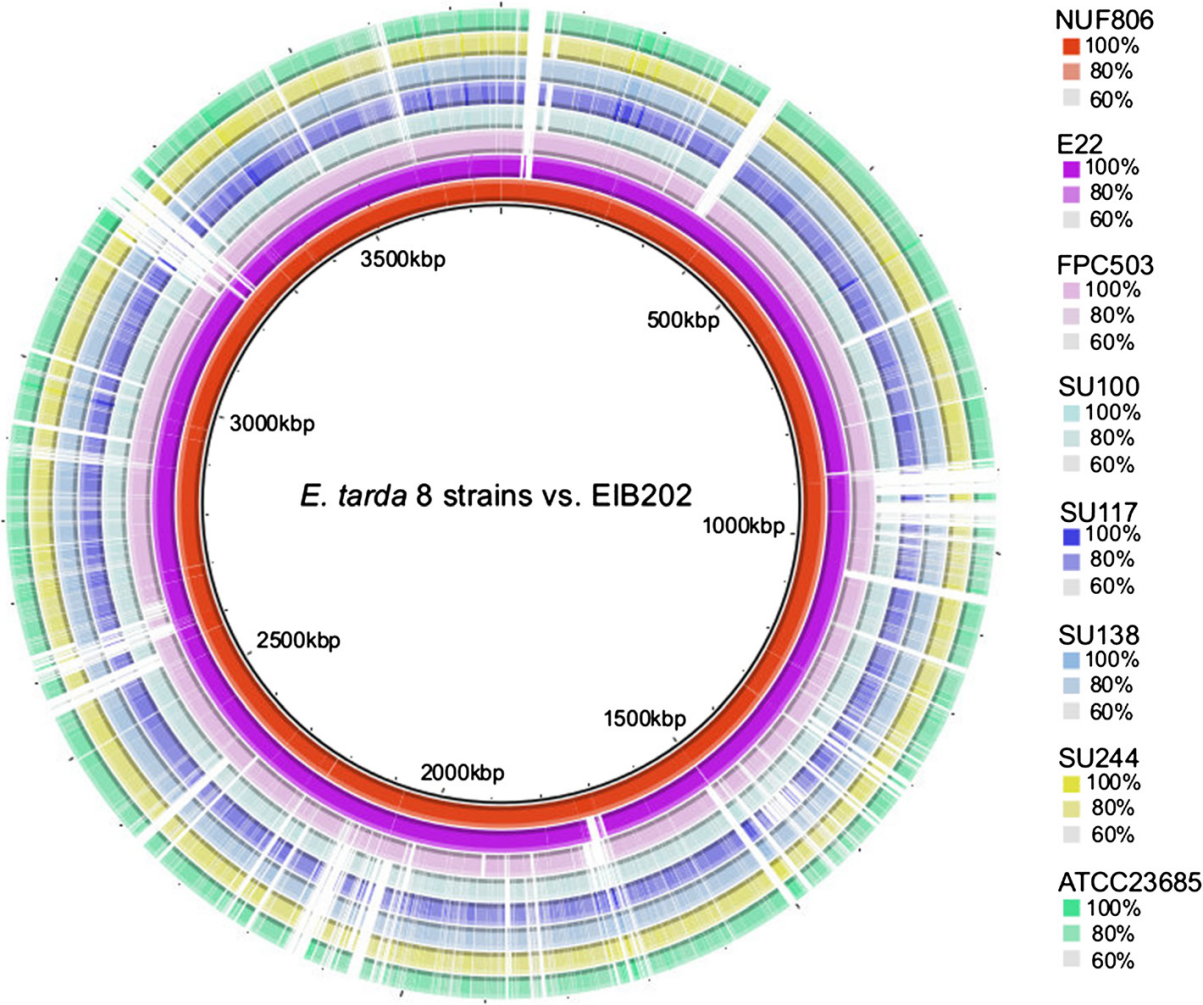
**Comparison of genome structure among *E. tarda *strains.** The genome contigs of the eight strains of *E. tarda* sequenced in this study were mapped to the genome of strain EIB202. The BLAST-based ring image was generated by BRIG [26].

To evaluate the assembly statistics, we resequenced the public *E. tarda* strain ATCC23685 in parallel with the other seven *E. tarda* strains, and compared the data (Additional file 3: Figure S3). For ATCC23685, we obtained 123 contigs consisting of 3,655,430 bp by *de novo* assembly; the public sequence had 87 contigs consisting of 3,744,568 bp. A total of 3,605,608 bp (98.6%) of the 3,655,430 bp mapped to the public scaffold sequence, and more than 99.9% of mapped nucleotides were identical. We compared the average identity of all the sequenced genomes among all the strains of this study, and found that the fish-pathogenic and environmental strains were clearly different from each other at the sequence similarity level (Table 3). The nucleotide sequence of the FPC503 (from red sea bream) was similar to the NUF806 (flounder) and E22 (eel) sequences, but differed by about 5%. Using the genome sequence of strain EIB202 as the reference, we compared the genomic structure among the eight strains by contig mapping (Figure 1). We found that the EIB202 genome was covered almost entirely by the contigs of NUF806 and E22, but some loci in the EIB202 genome were absent in the other six strains. Indeed, the EIB202, NUF806 and E22 genomes are highly similar at the sequence level (Table 3), indicating that, of the eight strains, these three strains are the most closely related.

**Table 3:** **Sequence similarity among *E. tarda *** **strains**

**Strain**	**NUF806**	**E22**	**FPC503**	**SU100**	**SU117**	**SU138**	**SU244**	**ATCC23685 (this study)**	**EIB202**	**ATCC23685 (public)**
NUF806	-	99.94	94.92	82.92	82.91	82.97	82.90	83.00	99.99	83.06
E22	-	-	94.91	82.94	83.06	83.01	83.08	83.01	99.94	83.05
FPC503	-	-	-	83.06	83.12	83.17	83.12	83.08	94.92	83.11
SU100	-	-	-	-	98.95	96.87	98.75	98.00	82.98	98.01
SU117	-	-	-	-	-	96.88	99.55	98.09	82.98	98.09
SU138	-	-	-	-	-	-	97.10	97.07	82.98	97.07
SU244	-	-	-	-	-	-	-	97.97	82.93	97.98
ATCC23685	-	-	-	-	-	-	-	-	83.02	99.98

Average sequence similarity percentages (length >= 100bp) are shown.

### Gene prediction and validation

We detected 3400–3900 ORFs in the sequenced *E. tarda* strains (Table 2). Of these predicted genes, an average of 96% (3258–3759 genes, excluding ATCC23685) matched known sequences. For ATCC23685, we predicted a smaller number of ORFs (3434 genes) than was predicted in the public reference data (3964 genes); 3276 of the genes were common to both sets of data as predicted by BLASTP. One reason why the gene numbers are different between the two sets of ATCC23685 sequence data might be inaccuracy in genome assembly. The ATCC23685 sequence obtained in this study has more contigs (123 contigs) and a shorter average length than the reference sequence (Table 2), implying that genes split by gaps between contigs have been missed by the gene-finding software. Another feasible reason may be that the reference data are of low quality. We checked the reference gene annotations and found that 302 genes have incorrect lengths (indivisible by three), suggesting that some of the reference genes are either pseudogenes or have been overestimated by false-positives (Additional file 4: Figure S4). Using mutual TBLASTN to query the protein sequences against the contig sequences, we were able to find almost all of the missing genes in each ATCC23685 sequence. Finally, we confirmed that a total of 3426 (99.8%) genes in our sequence were also present in the reference sequence, and 3934 (99.2%) genes in the reference sequence were present in our ATCC23685 sequence. Thus, we concluded that the genome data of the *E. tarda* strains of this study covered more than 99% of protein-coding loci and are accurate enough to be further compared.

### Gene comparison

To detect genetic differences between the *E. tarda* strains, we focused first on SNPs and INDELs. We mapped the NUF86 and E22 reads to the turbot pathogen strain EIB202 genome, because we had found that the sequences were highly similar to each other (Figure 1 and Table 3). We predicted a total of 79 SNPs or INDELs between NUF806 and EIB202, and 355 between E22 and EIB202 (Additional file 5: Table S1). Although most of the detected SNPs or INDELs were located in non-coding regions, 40 and 242 SNP/INDEL candidates were in the coding regions in NUF806 and E22, respectively. In this study, we focused on nonsense or frameshift mutations in protein-coding genes (Table 4), because such mutations are more likely to result in loss of function of the proteins that they encode. We found nine genes in E22 and only two genes in NUF806 that contained loss-of-function mutations. In particular, E22 had a nonsense mutation in the *esrB* of T3SS, which is involved in the virulence of *E. tarda*[27]. Because the E22 strain has been attenuated during cultivation, a few mutations may have occurred in a short period. We propose, therefore, that the mutation in *esrB* may be responsible for the attenuation of this strain.

**Table 4:** **Loss-of-function mutation in *E. tarda *** **strains E22 and NUF806**

**Gene product**	**Gene**	Length (bp)*	**Nonsynonymous mutation**	**INDEL**	**Type**
*Strain E22*					
hypothetical protein	ETAE_0831	1668	TGG(W)998TAG(*)		Nonsense
two-component response regulator	*esrB*	645	TCG(S)374TAG(*)		Nonsense
putative NADH:flavin oxidoreductase/NADH oxidase	ETAE_0969	2067	TGT(C)1116TGA(*)		Nonsense
ferric enterobactin transport protein	*fepE*	1047	AAC(N)134AGC(S)	GCGGC992	Frameshift
phospholipase D family protein	ETAE_1290	1245		G880	Frameshift
putative exoprotein-precursor	ETAE_2088	825		G809	Frameshift
bifunctional glutathionylspermidine	ETAE_2689	348	GCG(A)331TCG(S)	332CGCCGGT	Frameshift
cobyrinic acid ac-diamide synthase	ETAE_2747	132		C119	Frameshift
bifunctional chorismate mutase/prephenate dehydrogenase	*tyrA*	1122		C524	Frameshift
*Strain NUF806*					
hypothetical protein	ETAE_0339	120		78T	Frameshift
dihydropyrimidinase	*ygeZ*	1386		962G	Frameshift

* Lengths of EIB202 genes.

**Figure 2:**
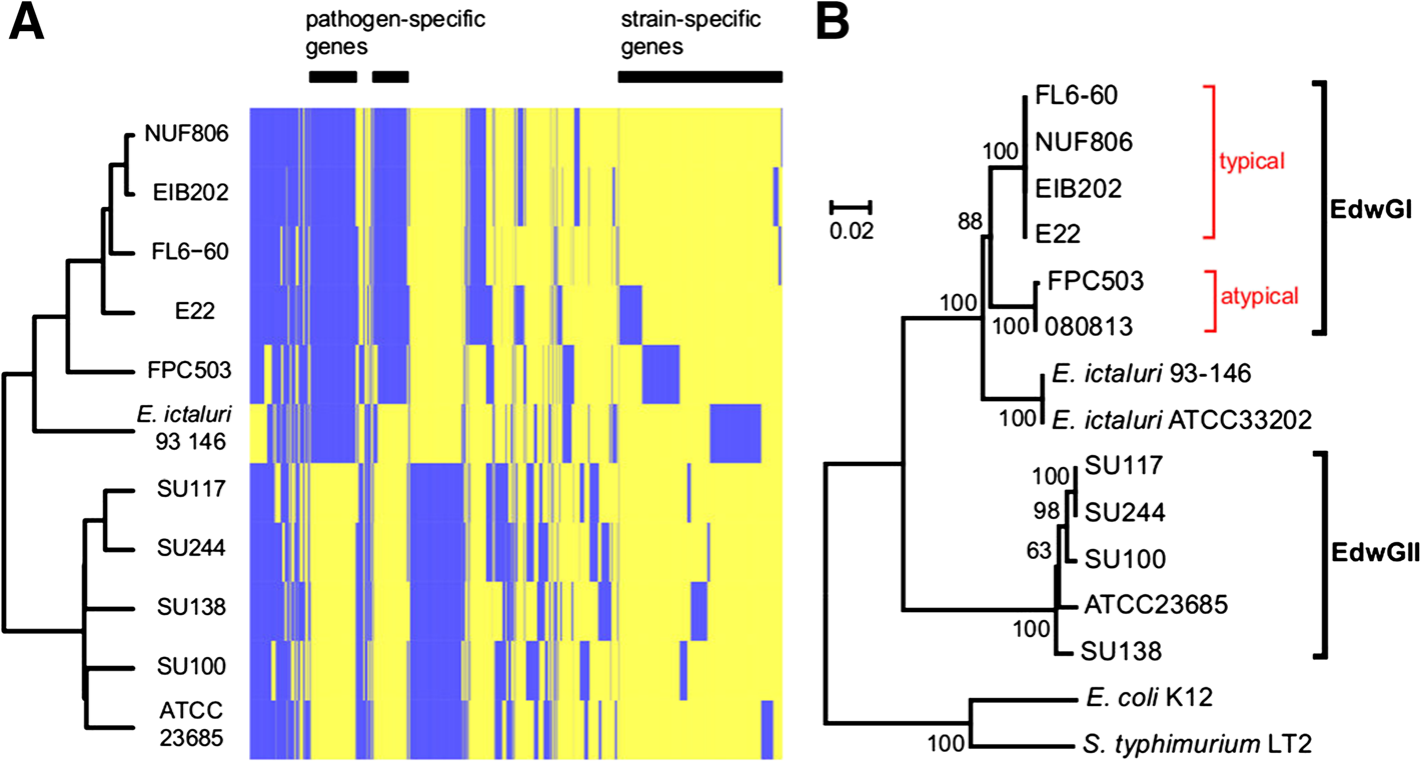
**Polymorphism of non-core genes among *E. tarda *strains. (A)** Map of polymorphic genes that are either present or absent among the strains. The presence/absence of genes is shown in blue/yellow, respectively. **(B)** Phylogenetic tree of DNA gyrase subunit B genes (*gyrB*).

We performed an all-vs-all BLASTP using the gene sets of eleven *Edwardsiella* strains (NUF806, E22, FPC503, SU100, SU117, SU138, SU244, and public *E. tarda* strains EIB202, FL60, ATCC23685, and *E. ictaluri* 93–146). We found that at least 2422 genes were conserved among all the strains, and 4147 genes were polymorphic, that is, each gene was absent from one or more of the eleven strains. We converted the polymorphism (presence/absence) of genes into a distance matrix and conducted cluster analysis. The dendrogram that we obtained was congruent with the molecular phylogenetic trees (Figure 2), suggesting that gene gain/loss events reflect the evolutionary scenario of the *Edwardsiella* lineage. In particular, the gene catalogues of the fish pathogen and non-pathogen strains were clearly distinct from each other, consistent with the previous study [14]. In this topology, *E. ictaluri* was positioned between pathogenic and environmental *E. tarda*, suggesting that the classification and nomenclature of *Edwardsiella* species may need to be reconsidered [28]. Moreover, all the serotype A strains, the typical (NUF806 and E22) and the atypical (FPC503), were classified into a single genotype EdwGI; the other serotype strains were clustered with ATCC23685, which has an EdwGII genotype (Figure 2B). It should be noted that FPC503 constituted a different clade from that of the typical serotype A strains, suggesting that the EdwGI group may be composed of two subgroups.

**Figure 3:**
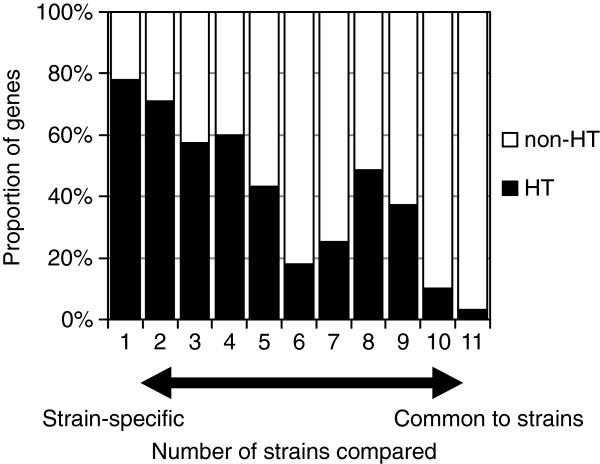
**Distribution of horizontally transferred genes specific/common to *E. tarda *strains.** The black bars indicate the proportions of horizontally transferred (HT) genes. The one at ‘11’ indicates the proportion of HT genes out of the genes common to all the strains. The one at ‘1’ indicates the proportion of HT genes out of the strain-specific genes.

To investigate the origin of the polymorphic genes among *E. tarda* strains, we conducted a horizontal gene transfer analysis (Figure 3). We found that most of the strain-specific genes tended to be horizontally transferred (HT), while most of the common genes were non-HT genes. Interestingly, the proportion of HT genes dropped around six strains as shown in Figure 3. This result can be explained by our experimental design: six fish-pathogens (NUF806, E22, FPC503, EIB202, FL6-60, and *E. ictaluri* 93–146) and five non-fish-pathogens (SU100, SU117, SU138, SU244, and ATCC23685), which corresponded to two phylogenetically distinct clades (as described above), were used in the study. Thus, the observed paucity of HT genes around six strains probably reflects clade-specific loss events of ancestral genes. One may speculate that the HT genes detected in this study may be artifacts due to DNA contamination in sequencing. However, we note that the HT genes common to *E. tarda* strains were distributed preferentially to either of the two clades (Additional file 6: Figure S5), likely reflecting the gene gain events in each lineage [14]. In addition, many (121/323) of strain-specific HT genes annotated were mobile element genes, such as phage-, plasmid, or transposon-related ones, which is unexplainable by DNA contamination. The presence/absence of virulence genes in *E. tarda* is summarized in Table 5 (Additional file 7: Table S2) [2,3]. Fish-pathogenic strains have two secretion system genes (T3SS and T6SS) and pilus assembly genes. We predicted that the T3SS and T6SS genes are both non-HT genes, while the pilus assembly genes are HT genes. We concluded that the T3SS and T6SS genes originated in an ancestral *Edwardsiella* lineage and were subsequently lost in non-pathogenic *E. tarda*[14]. However, here we noted that a gene in the T6SS locus, *evpP*, was predicted as being an HT gene. The *evpP* gene is located at the end of the T6SS locus; therefore, it may have been added to the locus after the divergence of pathogenic- and non-pathogenic *E. tarda*[29]. Particularly, it has been shown that deletion of *evpP* in *E. tarda* significantly decreased the virulence of the pathogens in fish [29]. Here, we propose that the ancestral T6SS of the *Edwardsiella* lineage was not originally involved in pathogenesis and that the subsequent acquisition of *evpP* contributed to the virulence of *E. tarda*. We also compared the genes related to biosynthesis of lipopolysaccharides as O-antigens among the *E. tarda* strains, and found polymorphisms related to the presence/absence of *rfb* homologs [30] (Additional file 8: Table S3), possibly due to horizontal transfer. The serotype A strains (NUF806, E22 and FPC503) share all the genes reported in *E. tarda* EIB202, which is characteristic of genotype EdwGI [14]. Non-pathogenic strains (SU100, SU117, SU138 and SU244) are different from the serotype A strains and also from each other. This presence/absence of *rfb* polymorphism might explain why non-pathogenic strains have different serotypes (B to D).

**Table 5:** **Comparison of reported virulence genes among *E. tarda *** **strains**

**Gene or gene cluster**	**Description**	**Accession No.**	**Reference**	**HT**^**†**^	**EIB202**	**NUF806**	**FL6-60**	**E22**	**FPC503**	**93_146**	**ATCC23685**	**SU100**	**SU117**	**SU138**	**SU244**
*hhaEt*	α-hemolysisn-modulator like protein	YP_003295064	[10]	X^§^	+	+	+	+	+	+	+	+	+	+	+
*papA*	P pilus assembly protein, pilin FimA	YP_003296301	[10]	X	+	+	+	+	+	-	-	-	-	-	-
*papC*	P pilus assembly protein, porin PapC	YP_003296298	[10]	X	+	+	+	+	+	?	?	?	?	?	?
*papD*	chaperone protein PapD	YP_003296297	[10]	X	+	+	+	+	+	?	?	?	?	?	?
*papH*	minor pilin subunit PapH	YP_003296299	[10]	X	+	+	?	+	+	-	-	-	-	-	-
*papK*	hypothetical protein	YP_003296296	[10]	X	+	+	?	+	+	-	-	-	-	-	-
*aidA*	putative autotransporter protein	BAH03175	[15]		-	-	-	-	+	+	-	-	-	-	-
*sodB*	iron-containing superoxide disumutase	BAA84480	[31]		+	+	+	+	+	+	+	+	+	+	+
*ethA*	hemolysin	BAA21097	[32]		+	+	+	+	+	+	+	+	+	+	+
*ethB*	activation/secretion protein	BAA21096	[32]		+	+	+	+	+	+	+	+	+	+	+
*qseB*	DNA-binding transcriptional regulator	ADO13165	Direct submission		+	+	+	+	+	+	+	+	+	+	+
*qseC*	sensor protein	ADO24152	[33]		+	+	+	+	+	+	+	+	+	+	+
*phoP*	phoP	ADB28435	[34]		+	+	+	+	+	+	+	+	+	+	+
*phoQ*	phoQ	ADB28436	[34]		+	+	+	+	+	+	+	+	+	+	+
*phoU*	repressor protein	AAN05785	[35]		+	+	+	+	+	+	+	+	+	+	+
*fimABCD*	type 1 fimbrial protein	BAC55512-3, BAD00163-4	[35,36]		+	+	+	+	+	+	+	+	+	+	+
*ankB*	ankyrin B	AAL82720	[35]	X	+	+	?	+	+	-	-	-	-	-	-
*citC*	citrate lyase ligase	AAO52821	[35]		+	+	+	+	+	+	+	+	+	+	+
*gadB*	glutamate decarboxylase	AAL82718	[35]		+	+	+	+	+	+	+	+	+	+	+
*isoR*	Fe-S oxidoreductase	AAL82723	[35]		+	+	+	+	+	+	+	+	+	+	+
*katB*	catalase precursor	AAL82719	[35]	X	+	+	+	+	+	-	-	-	-	-	-
*mukF*	putative killing factor	AAL82725	[35]		+	+	+	+	+	+	+	+	+	+	+
*ompS2*	outer membrane protein	AAL82724	[35]		+	+	+	+	+	+	+	+	+	+	+
*orf20*	hypothetical protein	AAL82721	[35]		-	-	-	-	-	-	-	-	-	-	-
*orfA*	unknown protein	AAL01251	[35]		+	+	+	+	+	+	-	-	-	-	-
*pstA*	transport membrane protein A	AAN05783	[35]		+	+	+	+	+	+	+	+	+	+	+
*pstB*	ATP binding protein	AAN05784	[35]		+	+	+	+	+	+	+	+	+	+	+
*pstC*	transport membrane protein C	AAN05782	[35]		+	+	+	+	+	+	+	+	+	+	+
*pstS*	phosphate binding protein	AAO52825	[35]		+	+	+	+	+	+	+	+	+	+	+
*flagellin*	flagellin	AAN52540	[37]		+	+	+	+	+	+	+	+	+	+	+
*esa, esc, ese, esr*	Type III secretion system loci	AAV69401- AAX76924^*^	[37]		+	+	+	+	+	+	-	-	-	-	-
*evp*	Type VI secretion system locus	AAR83927 - ABW69087^*^	[38]	*evpP*^‡^	+	+	+	+	+	+	-	-	-	-	-

Denotation: ‘+’, present (amino acid identity >= 60%); ‘-’, absent; ‘?’, weekly similar (amino acid identity < 60%).

*All accession numbers are shown in Additional file 7: Table S2.

^†^Whether horizontally transferred or not was predicted.

^§^HT was predicted in fish-pathogens.

^‡^A gene *evpP* in this locus was predicted as horizontally transferred.

### NUF806-specific genes

Among the eight sequenced strains in this study, we observed that NUF806 and EIB202 were the closest at the genome sequence level; almost all the genes were common to both strains. However, unlike EIB202, NUF806 lacked plasmid-encoding genes, namely, the type IV secretion system (T4SS) that is involved in conjugative transfer of plasmid, and the drug-resistance genes against streptomycin and chloramphenicol. Therefore, NUF806 may be sensitive to these antibiotics. Because NUF806 and EIB202 are flounder pathogens with similar virulence, this finding suggested that the plasmid-encoding genes are not essential for pathogenesis in flounder.

### E22-specific genes

Among the eight strains in this study, E22 is the second closest strain to EIB202. Although there were no major differences in the gene sets of the two strains, we found that loss-of-function mutations had occurred in some of the genes (Table 4). On the other hand, we found that E22 had plasmid-related genes which were almost identical to corresponding genes in a conjugative plasmid (pRA1) isolated from a fish-pathogenic bacterium, *Aeromonas hydrophila*[39]. The plasmid genes were encoded in four contigs with a total length of 140 kb, which covered more than 90% of the pRA1 genome (Additional file 9: Figure S6). Because the gene that encodes RepA (plasmid replication protein) and conjugative transfer genes were included in the region, the contigs probably constitute an intact plasmid which is not integrated into the E22 chromosome. The plasmid of E22 also carries drug-resistance genes, *tetRA* for tetracycline, *sul2* for sulfonamides, and *hipAB* for beta-lactams. Previously, it was reported that many of the pathogenic *E. tarda* strains isolated from eel were resistant to tetracycline and sulfamonomethoxine, probably because of continued drug treatment in eel ponds [40]. The previous study had demonstrated that such drug-resistance markers may be located on an 81-kb conjugative plasmid [40]. We propose that the longer E22 plasmid is evolutionarily related to the previously reported 81-kb conjugative plasmid, and that these may share a common ancestor with the plasmids isolated from *A. hydrophila*[41].

### FPC503-specific genes

**Figure 4:**
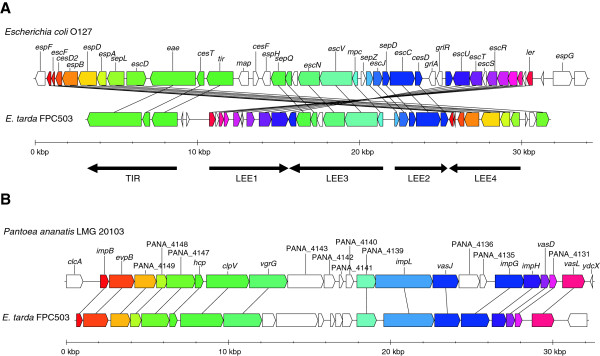
**Novel pathogenicity islands of *E. tarda *FPC503.** The syntenies of the T3SS and T6SS genes were compared with the corresponding genes in *Escherichia coli* O127 and *P. ananatis*, respectively. Orthologous genes are in the same color and are linked by lines. **(A)** The T3SS locus (Et-LEE). The positions of five major operons (LEE1, LEE2, LEE3, LEE4, and TIR) are shown below the panel. **(B)** The T6SS locus (Et-T6SS2).

We found that FPC503 had genes of the novel T3SS and T6SS which are not present in the other *E. tarda* strains in this study. These genes were predictable in strain 080813 which is a close relative of FPC503 (Figure 2), although the contigs of 080813 are still fragmented (T3SS, [GenBank:AFJH01000035]; T6SS, [GenBank:AFJH01000029]). Therefore, the second T3SS and T6SS were considered to be a common feature of the atypical *E. tarda*, which is distinct from the typical strains. At the sequence level, the second T3SS was similar to the T3SS of *E. coli*, and the T6SS was similar to the T6SSs in other enterobacteria, *Enterobacter* and *Pantoea*. To examine the locus structures in detail, we sequenced the genome of FPC503 using longer-read 454 pyrosequencing. *De novo* assembly produced a single contig for the T3SS locus, and two contigs for the T6SS which were further joined into a single contig by PCR-based genome walking. Both contigs contained, at either end, the genes that were present in the *E. tarda* EIB202 chromosome, implying that these contigs were derived from the FPC503 chromosome and not from the plasmids. We observed that homologs of intimin [42] and Tir (translocated intimin receptor) [43] were encoded in the T3SS cluster. These genes (*eae* and *tir*) are known to be important elements in a pathogenicity island of enteropathogenic and enterohemorrhagic *E. coli* strains, namely the locus of enterocyte effacement (LEE) [44]. Strikingly, when we compared the gene content and order between the FPC503 T3SS cluster and the *E. coli* LEEs, we found that they were well conserved (Figure 4A and Additional file 10: Figure S7A). Indeed, 29 out of 42 genes in enteropathogenic *E. coli* (and 28 out of 40 genes in enterohemorrhagic *E. coli*) were identified in the FPC503 T3SS locus, and the observed differences in the gene order were explainable by assuming a few recombination events. Furthermore, we observed microsynteny in each of the five major operons (LEE1, LEE2, LEE3, LEE4, and TIR), which constitute LEE [45]. Thus, we concluded that FPC503 had a LEE-like pathogenicity island that we named Et-LEE (*E. tarda* LEE). For the second T6SS, which we termed Et-T6SS2, we also observed a high synteny to a T6SS cluster in *P. ananatis* (Figure 4B). In particular, we found a homolog of *vgrG* that encodes an effector protein of T6SS [46]. As reported in other enterobacterial genomes [46,47], this gene is closely located to *hcp*, which was identified previously in *E. tarda*[15], suggesting that these genes may function as essential components of the Et-T6SS2 in FPC503. In the genome assembly of FPC503, we found another contigs that were similar to the Et-T6SS2 locus (Additional file 10: Figure S7B), implying that this locus was duplicated in FPC503.

It is known that pathogenicity-related genes often flow among species by horizontal gene transfer [21,48]. Using a Markov model method, we predicted that Et-LEE was extrinsic to FPC503 through recent horizontal transfer. The T6SS locus was not significantly predicted by the method, but the genes may possibly be of the horizontal origin because the gene sequences were highly similar to the corresponding genes in *Pantoea* (average amino acid identity = 80%) and no orthologs were present in other *E. tarda* strains. A difference between *E. coli* LEE and Et-LEE is their locations in the genomes: *E. coli* LEE was generally inserted next to a tRNA locus, but no tRNA locus was found close to Et-LEE. In addition, no transposable element related genes were detected near the Et-LEE, except for a member of the transposase IS3/IS911 family. Therefore, we proposed that Et-LEE may either have lost mobility after integration or have been inserted in a different manner than *E. coli* LEE.

**Figure 5:**
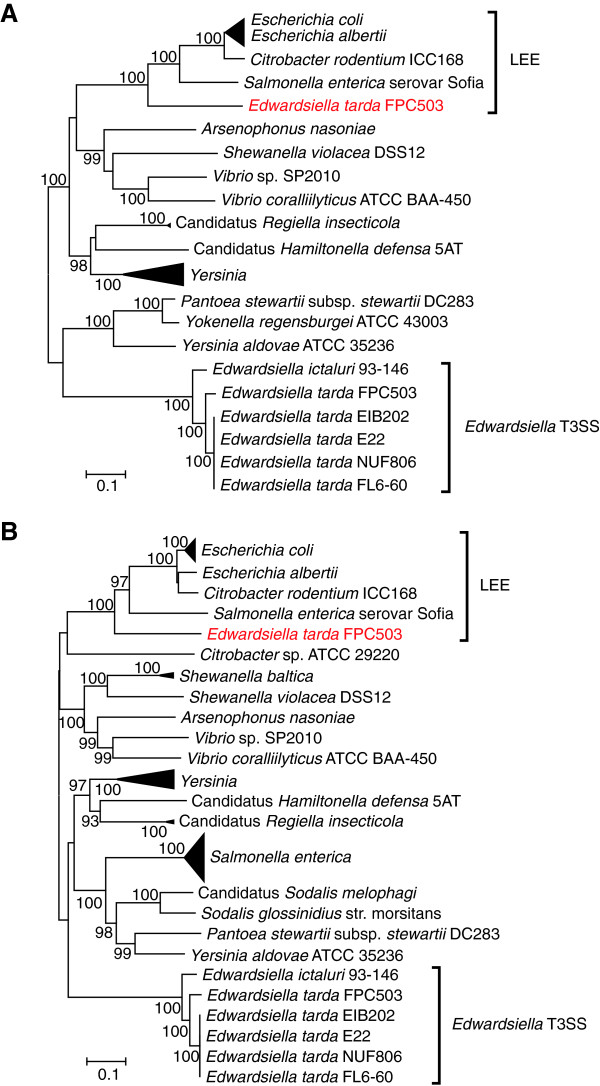
**Phylogenetic trees of T3SS genes.** Numbers at the branches indicate the bootstrap probabilities (≥90%) with 1000 replicates. **(A)** Phylogenetic tree based on the *escC* gene. **(B)** Phylogenetic tree based on the *escV* gene.

Our result raises a further question about why FPC503 acquired and retained Et-LEE. Since, in *E. coli*, the secreted Tir and intimin proteins encoded in LEE function in adhesion to intestinal epithelial cells [43,49-51], Et-LEE may also play a role in the intimate attachment of the pathogen to fish intestinal cell. We should keep in mind that FPC503 is a non-motile strain (Table 1), a trait that is disadvantageous for infection to host cells. Thus, a plausible explanation for the acquisition of Et-LEE by FPC503 may be that Et-LEE can compensate for its non-motility: when FPC503 is carried close to the host intestinal cells, it can fix tightly and effectively colonize its host by using Et-LEE. The origin of LEE in enterobacteria is also an unanswered question. LEE has been reported in pathogenic *E. coli*, in a mouse-pathogen *Citrobacter rodentium*[52], and in *Salmonella enterica*[53], but, until now, it has not been reported in fish pathogens. The current study has shown that the *E. tarda* strain that infects red sea bream may have also acquired Et-LEE by horizontal transfer, meaning that the donor species of LEE was not *E. tarda*. Molecular phylogenetic analysis indicated that all the Et-LEE genes examined were significantly close to the LEEs of *E. coli*, *C. rodentium* and *S. enterica* (Figure 5 and Additional file 11: Figure S8), suggesting that Et-LEE may be an appropriate outgroup of these LEEs. The sequencing of other *E. tarda* strains that harbor Et-LEE (e.g. strain 080813) may fill a missing link in the evolution of pathogenesis associated with LEE in enterobacteria.

## Conclusions

In this study, we determined the genome sequences of eight strains of *E. tarda* using next-generation sequencing technology. The GC content, hierarchical clustering based on gene repertoire, and phylogenetic tree, all clearly showed differences between the fish-pathogenic and environmental *E. tarda* genome sequences. By comparing the genomes, we identified polymorphisms that were responsible for serotypes and for the pathogenesis of *E. tarda*. We found that O-antigen related genes were different among each of the serotype strains, and that fish-pathogenic *E. tarda* was characterized by having two types of secretion systems (T3SS and T6SS) and pilus assembly genes. We predicted that the lineage- and species-specific genes may have originated by horizontal transfer, perhaps providing *E. tarda* with important traits that could be used as strain-dependent drug targets in aquaculture. Importantly, in this study, we found that the *E. tarda* strain that was isolated from red sea bream had T3SS (Et-LEE) and T6SS (Et-T6SS2) genes that were of horizontal origin from foreign organisms. This observation suggests that the previously proposed *E. tarda* genotype EdwGI could be divided into two sub-genotypes, a typical one and an Et-LEE/T6SS2-bearing (atypical) one. This is the first report that a fish pathogen possesses LEE, which is known in zoonotic pathogenic enterobacteria. This finding may provide a clue to the origin of the LEE pathogenicity island. Our results suggest that gene flow beyond species has a wide influence in the pathogenesis of enterobacteria.

### Availability of supporting data

The next-generation sequence data described in this article are available from the DDBJ Sequence Read Archive under accession ID DRA001012 at http://trace.ddbj.nig.ac.jp/DRASearch/submission?acc=DRA001012.

## Competing interests

The authors declare that they have no competing interests.

## Authors’ contributions

YN participated in the design of the study, performed the statistical analysis and drafted the manuscript. TT carried out the sample preparation and drafted the manuscript. MY carried out the genome sequencing and drafted the manuscript. TS and TM participated in the discussion of the study and revised the manuscript critically for important intellectual content. MS conceived of the study, and participated in its design. All authors read and approved the final manuscript.

## Supplementary Material

Additional file 1: Figure S1 Relationship between k-mer and N50 in *de novo* assembly. For each of the *E. tarda* strains, the N50 size of contigs produced is plotted versus the k-mer value chosen in the ABySS program [16]. Click here for file

Additional file 2: Figure S2 Relationship between k-mer and redundant contigs in *de novo* assembly. For each of the *E. tarda* strains, the redundant contigs size produced is plotted versus the k-mer value chosen in the ABySS program [16]. Click here for file

Additional file 3: Figure S3 Comparison of genome structure of *E. tarda* strain ATCC23685 between the sequenced and reference ones. The genome contigs of *E. tarda* ATCC23685 sequenced in this study were mapped to the reference genome [GenBank:ADGK01000000]. The BLAST-based ring image was generated by BRIG [26]. Click here for file

Additional file 4: Figure S4 Relationship between genome size and gene number. For each of the *E. tarda* strains, the gene number is plotted versus the genome size. The strain with the most genes (3934) is the public ATCC23685 [GenBank:ADGK01000000]. Click here for file

Additional file 5: Table S1 A summary of SNP and INDEL between *E. tarda* strains. Click here for file

Additional file 6: Figure S5. Distribution of horizontally transferred (HT) genes common to *E. tarda* strains. Seven strains (three fish-pathogens [NUF806, E22 and FPC503] and four non-pathogens [SU100, SU117, SU138, and SU244]) sequenced in this study were used. The black bars indicate the proportions of HT genes detected in only pathogenic strains. The gray bars indicate the proportions of HT genes detected in only non-pathogenic strains. The HT genes detected in both of the pathogenic and non-pathogenic strains are shown in white. Expected proportions were calculated by Monte Carlo simulation and the observed proportions were statistically significant (*p*<0.005). Click here for file

Additional file 7: Table S2 Accession numbers of virulence genes of *E. tarda*. Click here for file

Additional file 8: Table S3 O-antigen related genes among *E. tarda* strains. Click here for file

Additional file 9: Figure S6 Comparison of genome structure between the *Aeromonas hydrophila* plasmid pRA1 and the contigs obtained in the assembly of *E. tarda* strain E22. Four contigs out of those assembled for *E. tarda* E22 were mapped to the genome of *Aeromonas hydrophila* plasmid, pRA1 [39]. The BLAST-based ring image was generated by BRIG [26]. Click here for file

Additional file 10: Figure S7 Novel pathogenicity islands of *E. tarda* FPC503. The syntenies of the T3SS and T6SS genes were compared with the corresponding genes in *Escherichia coli* O157 and *P. ananatis*, respectively. Orthologous genes are in the same color and are linked by lines. (A) Et-LEE. (B) A possibly duplicated cluster of Et-T6SS2. Click here for file

Additional file 11: Figure S8 Phylogenetic trees of T3SS genes. Numbers at the branches indicate the bootstrap probabilities (≥90%) with 1000 replicates. Bracket indicates the clade of LEE genes. (A) Phylogenetic tree based on the *escJ* gene. (B) Phylogenetic tree based on the *escN* gene. (C) Phylogenetic tree based on the *escR* gene. Click here for file
