# Supplementary figures and images for: Comparative genomics reveals that a fish pathogenic bacterium Edwardsiella tarda has acquired the locus of enterocyte effacement (LEE) through horizontal gene transfer
Source: BMC Genomics. 2013 Sep 22;14:642. doi: 10.1186/1471-2164-14-642 (PMC3890536; doi:10.1186/1471-2164-14-642)

**Figure S1**

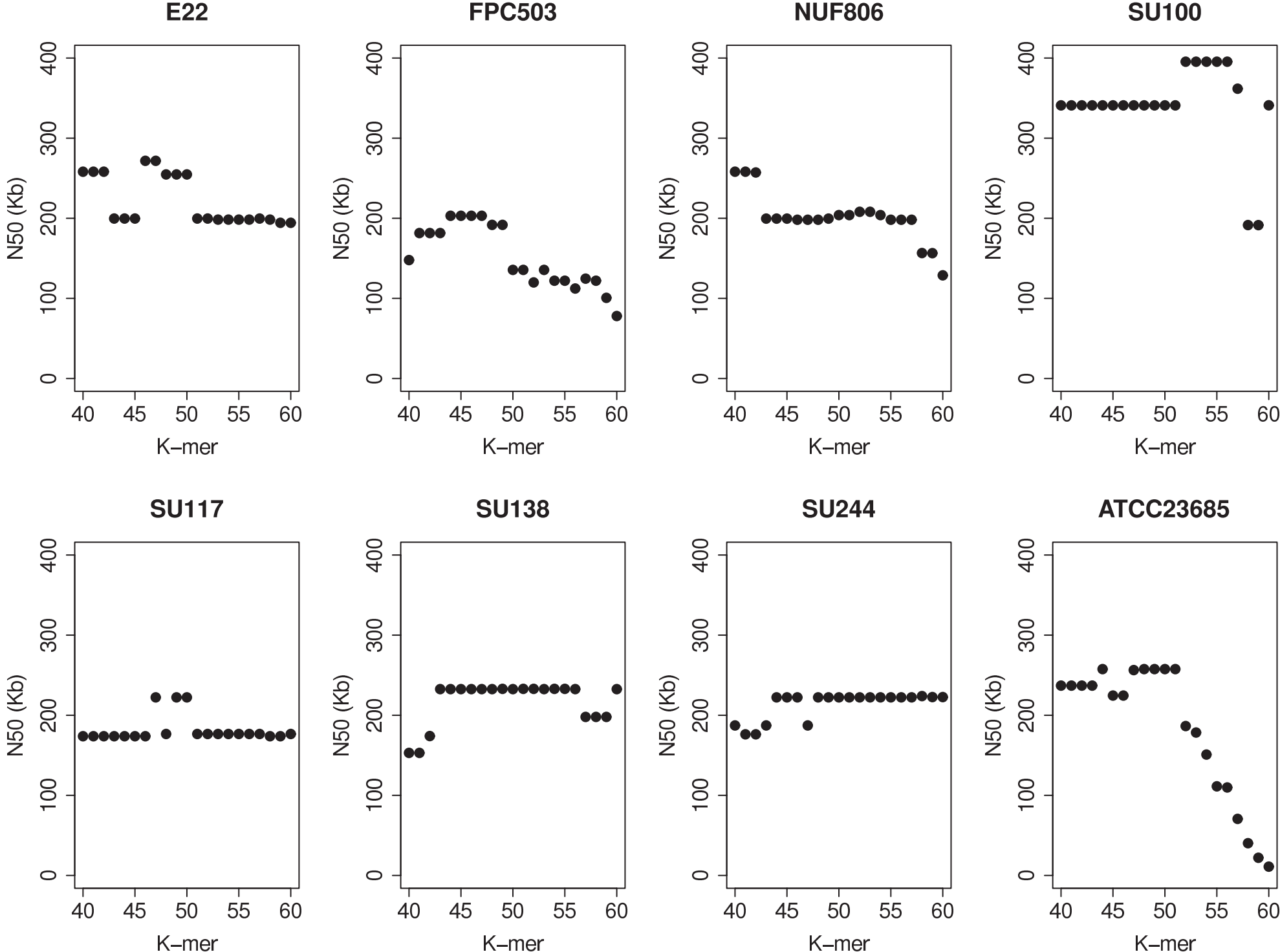

Supplement: Additional file 1: Figure S1 — Relationship between k-mer and N50 in de novo assembly. For each of the E. tarda strains, the N50 size of contigs produced is plotted versus the k-mer value chosen in the ABySS program [16]. [file 1471-2164-14-642-S1.pdf]

**Figure S2**

**E22**

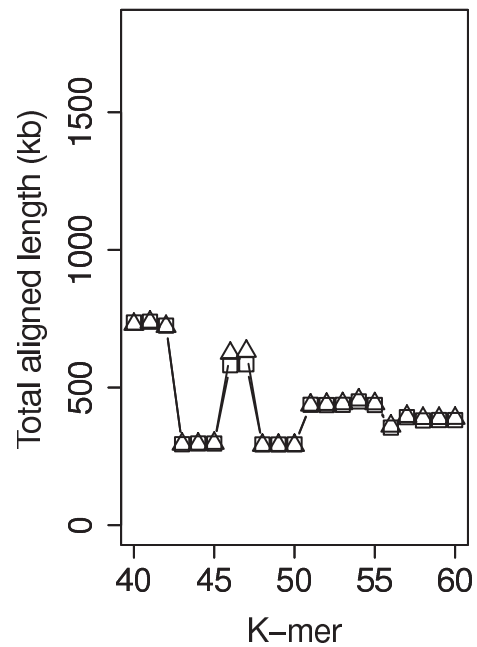

**FPC503**

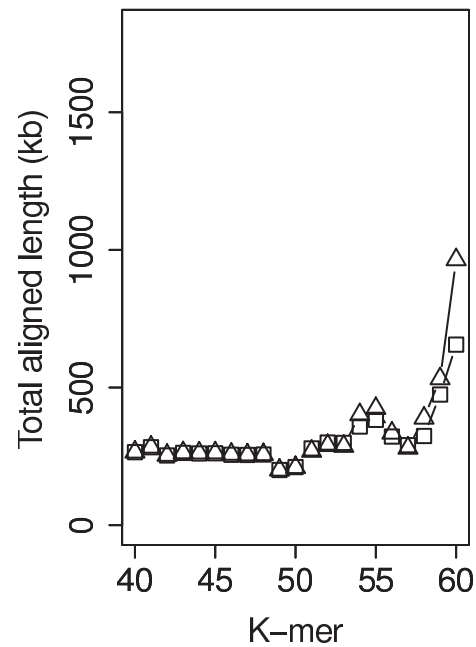

**NUF806**

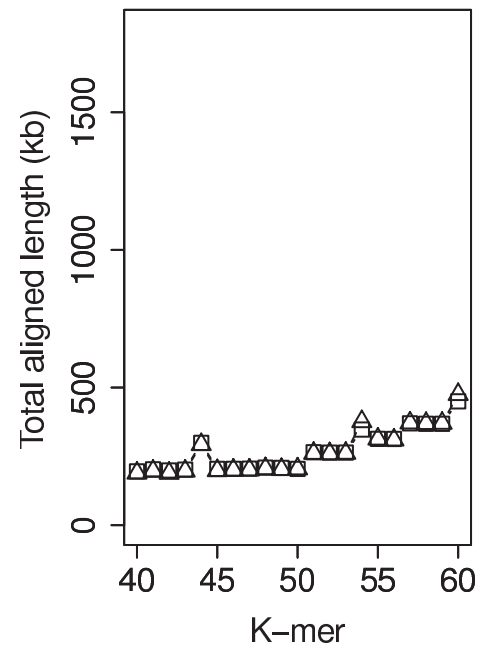

**SU100**

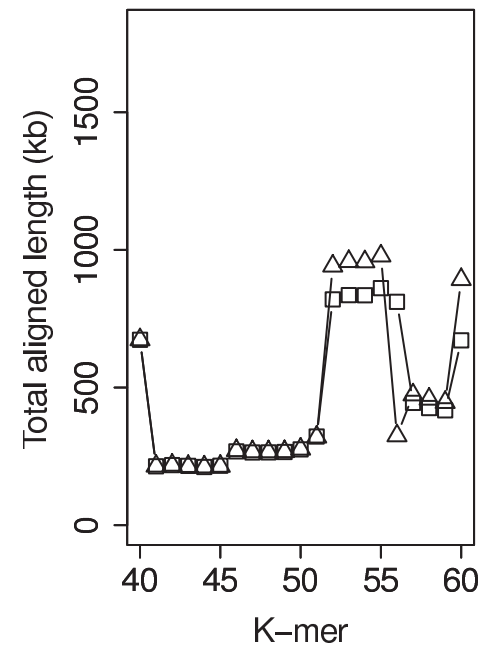

**SU117**

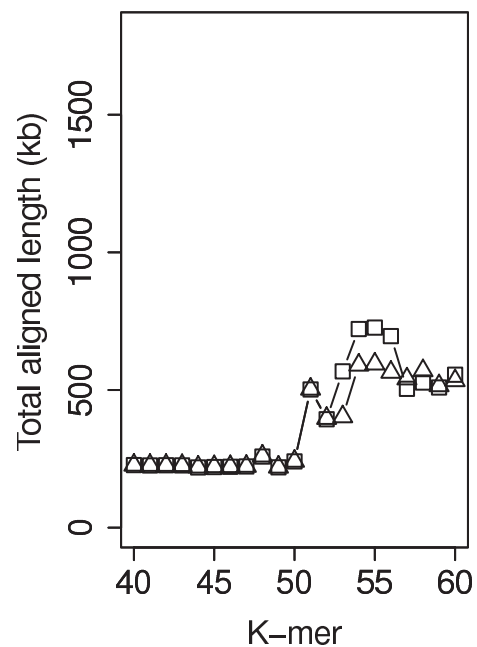

**SU138**

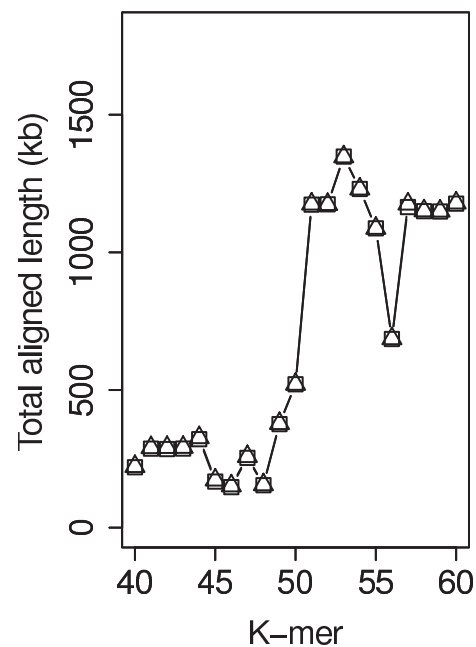

**SU244**

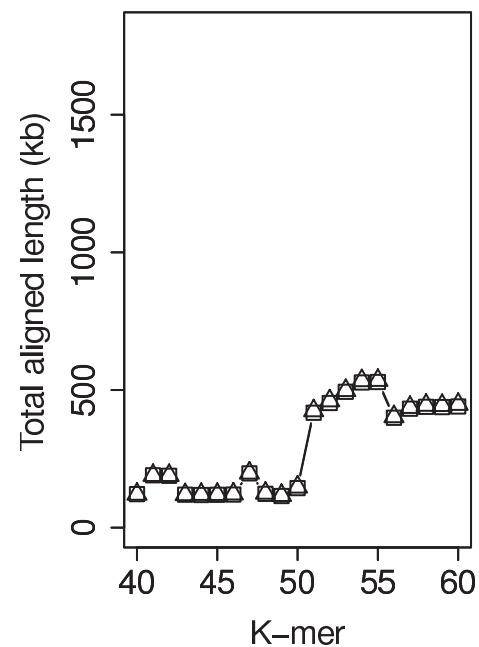

**ATCC23685**

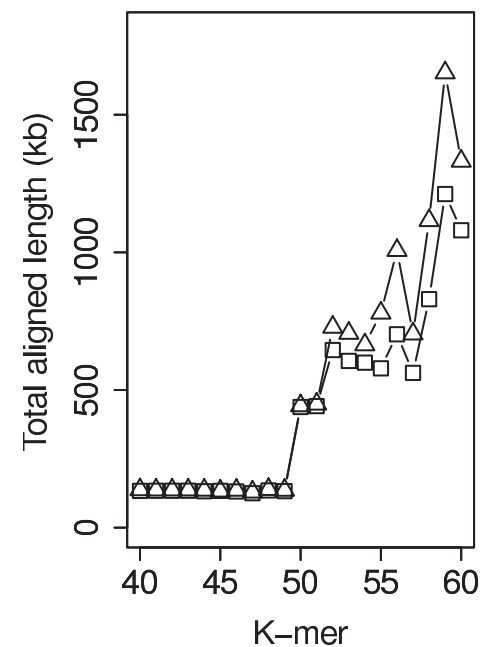

Supplement: Additional file 2: Figure S2 — Relationship between k-mer and redundant contigs in de novo assembly. For each of the E. tarda strains, the redundant contigs size produced is plotted versus the k-mer value chosen in the ABySS program [16]. [file 1471-2164-14-642-S2.pdf]

**Figure S3**

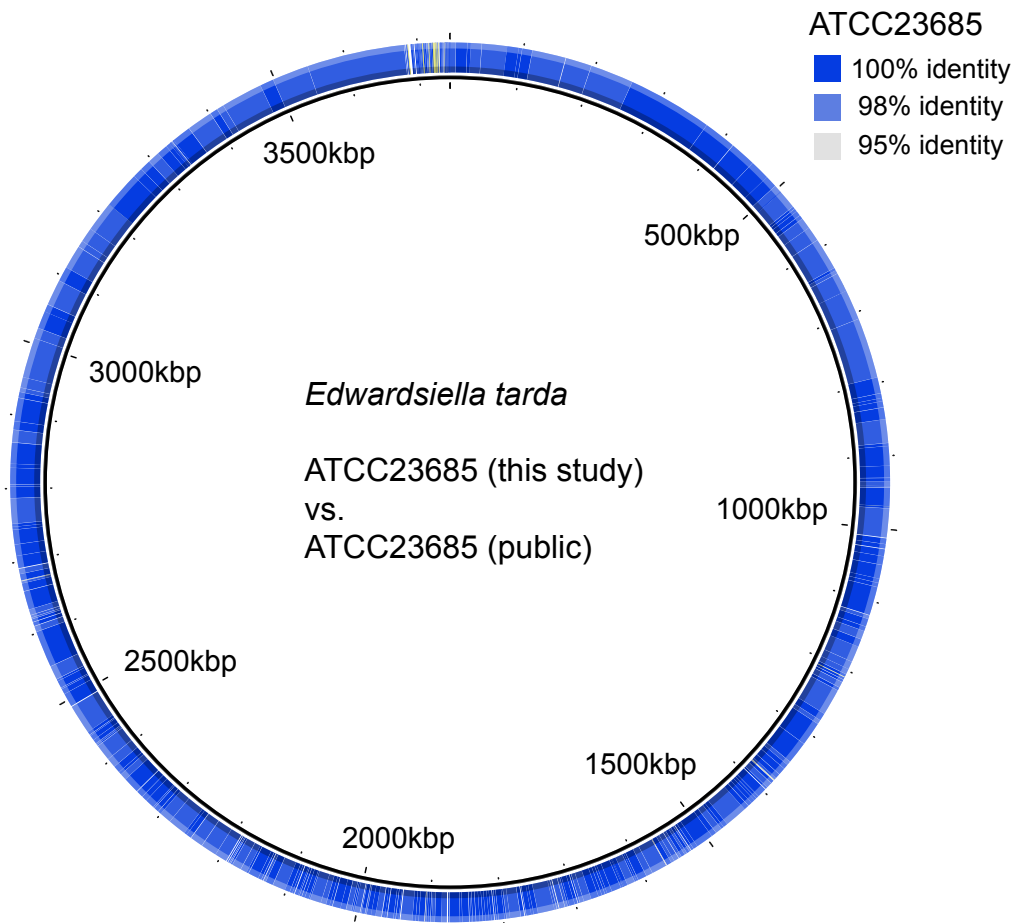

Supplement: Additional file 3: Figure S3 — Comparison of genome structure of E. tarda strain ATCC23685 between the sequenced and reference ones. The genome contigs of E. tarda ATCC23685 sequenced in this study were mapped to the reference genome [GenBank:ADGK01000000]. The BLAST-based ring image was generated by BRIG [26]. [file 1471-2164-14-642-S3.pdf]

**Figure S4**

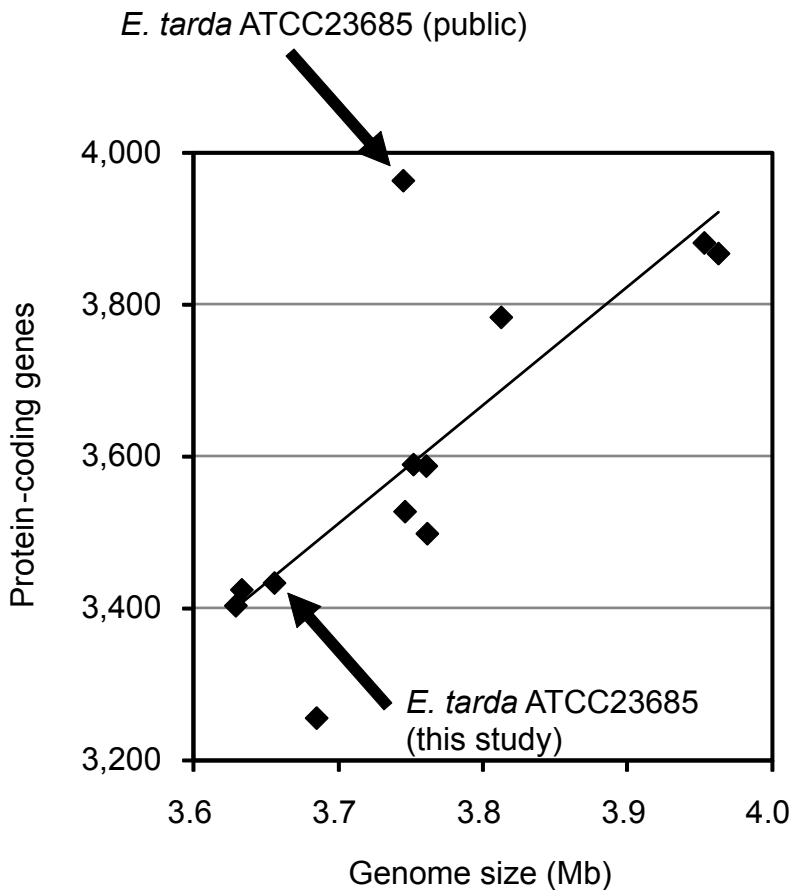

Supplement: Additional file 4: Figure S4 — Relationship between genome size and gene number. For each of the E. tarda strains, the gene number is plotted versus the genome size. The strain with the most genes (3934) is the public ATCC23685 [GenBank:ADGK01000000]. [file 1471-2164-14-642-S4.pdf]

**Figure S5**

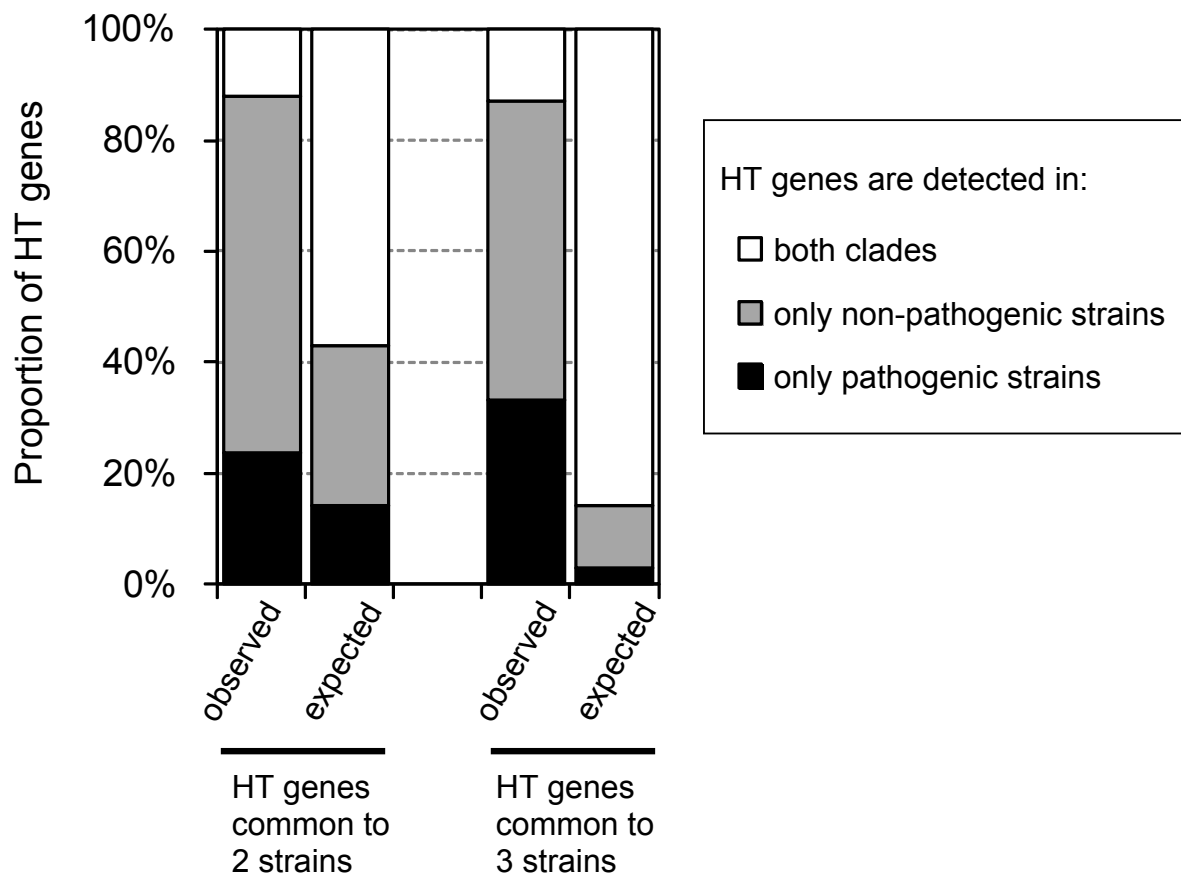

Supplement: Additional file 6: Figure S5. — Distribution of horizontally transferred (HT) genes common to E. tarda strains. Seven strains (three fish-pathogens [NUF806, E22 and FPC503] and four non-pathogens [SU100, SU117, SU138, and SU244]) sequenced in this study were used. The black bars indicate the proportions of HT genes detected in only pathogenic strains. The gray bars indicate the proportions of HT genes detected in only non-pathogenic strains. The HT genes detected in both of the pathogenic and non-pathogenic strains are shown in white. Expected proportions were calculated by Monte Carlo simulation and the observed proportions were statistically significant (p<0.005). [file 1471-2164-14-642-S6.pdf]

**Figure S6**

Four contigs of *E. tarda* E22  
vs. plasmid pRA1

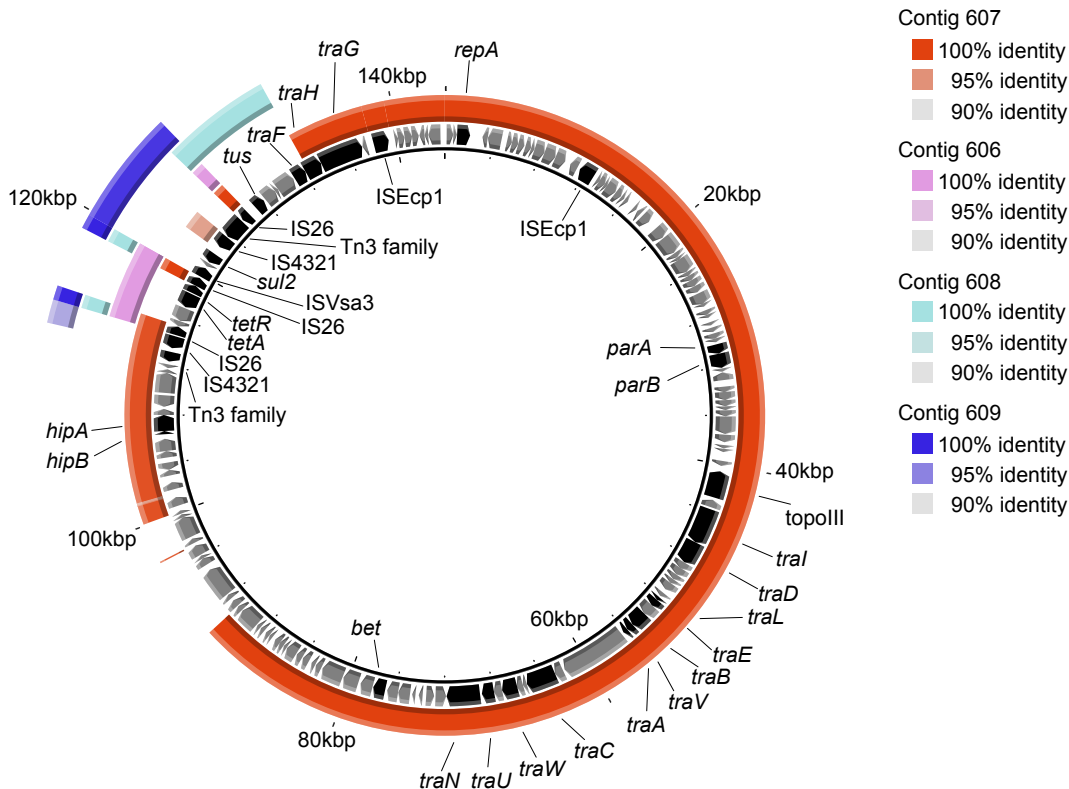

Supplement: Additional file 9: Figure S6 — Comparison of genome structure between the Aeromonas hydrophila plasmid pRA1 and the contigs obtained in the assembly of E. tarda strain E22. Four contigs out of those assembled for E. tarda E22 were mapped to the genome of Aeromonas hydrophila plasmid, pRA1 [39]. The BLAST-based ring image was generated by BRIG [26]. [file 1471-2164-14-642-S9.pdf]

Figure S7

A

*Escherichia coli* O157

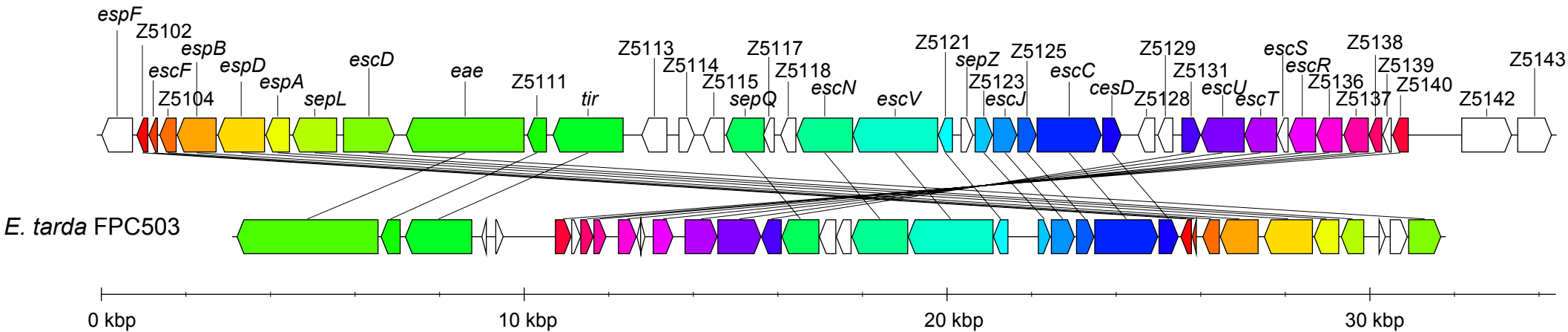

B

*Pantoea ananatis* LMG 20103

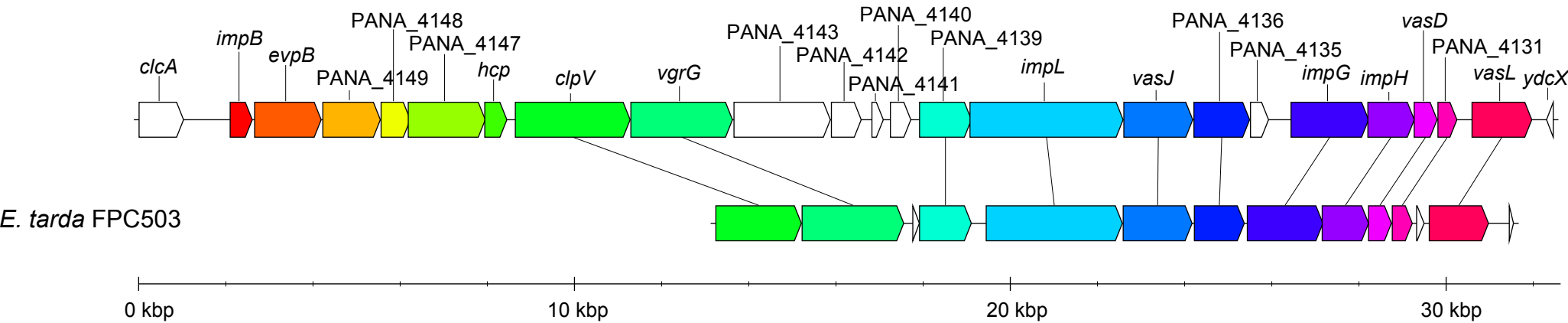

Supplement: Additional file 10: Figure S7 — Novel pathogenicity islands of E. tarda FPC503. The syntenies of the T3SS and T6SS genes were compared with the corresponding genes in Escherichia coli O157 and P. ananatis, respectively. Orthologous genes are in the same color and are linked by lines. (A) Et-LEE. (B) A possibly duplicated cluster of Et-T6SS2. [file 1471-2164-14-642-S10.pdf]

Figure S8

A

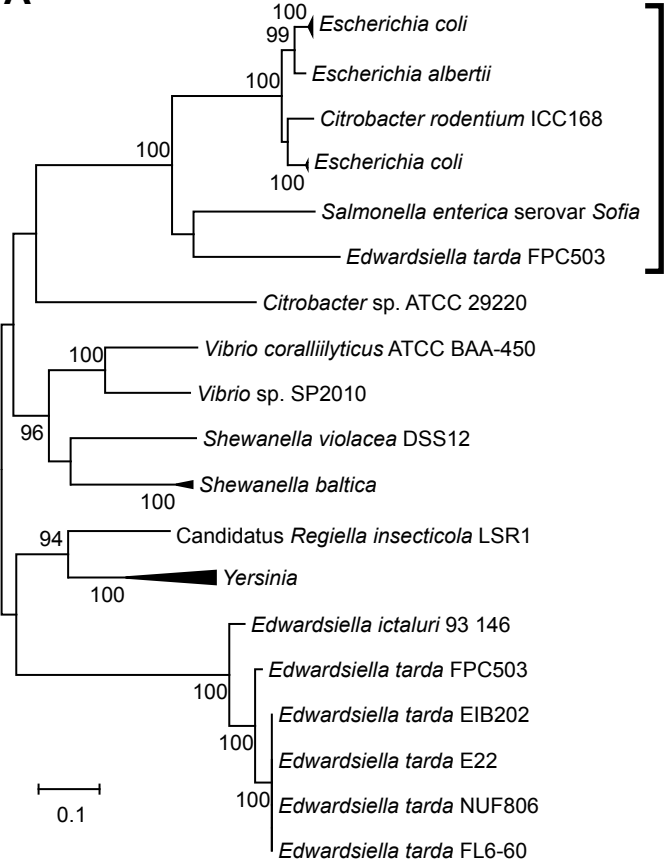

B

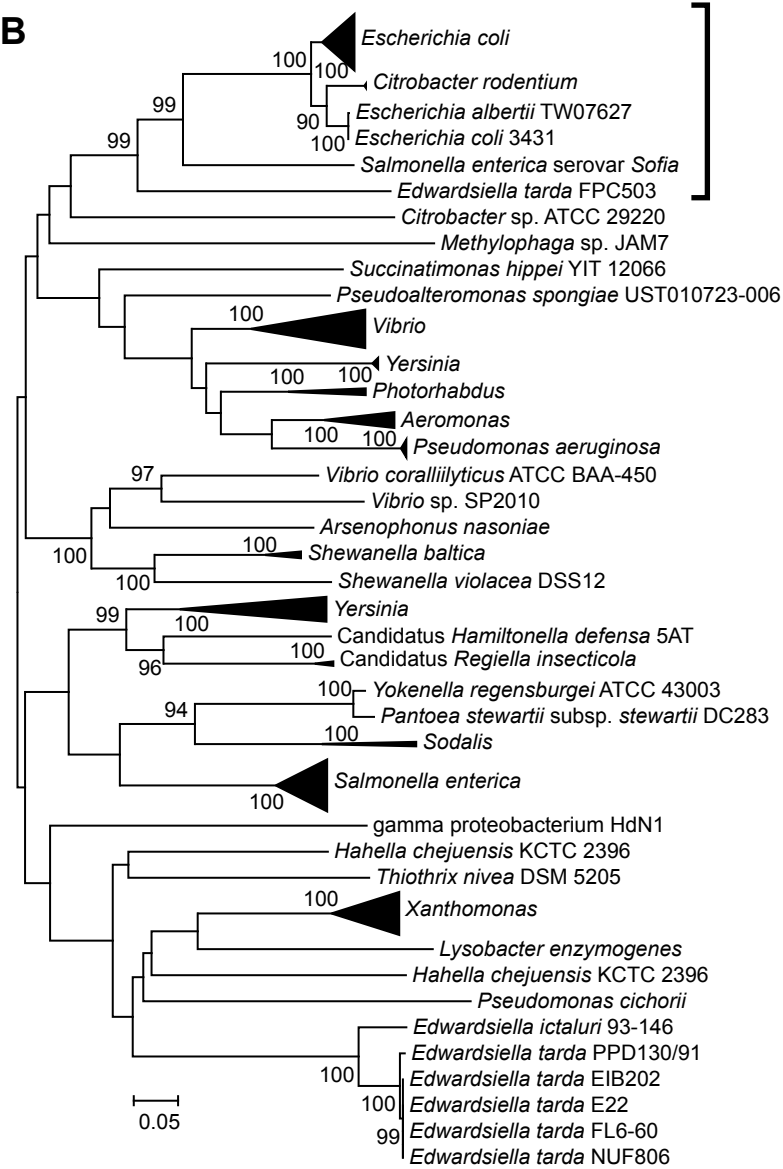

C

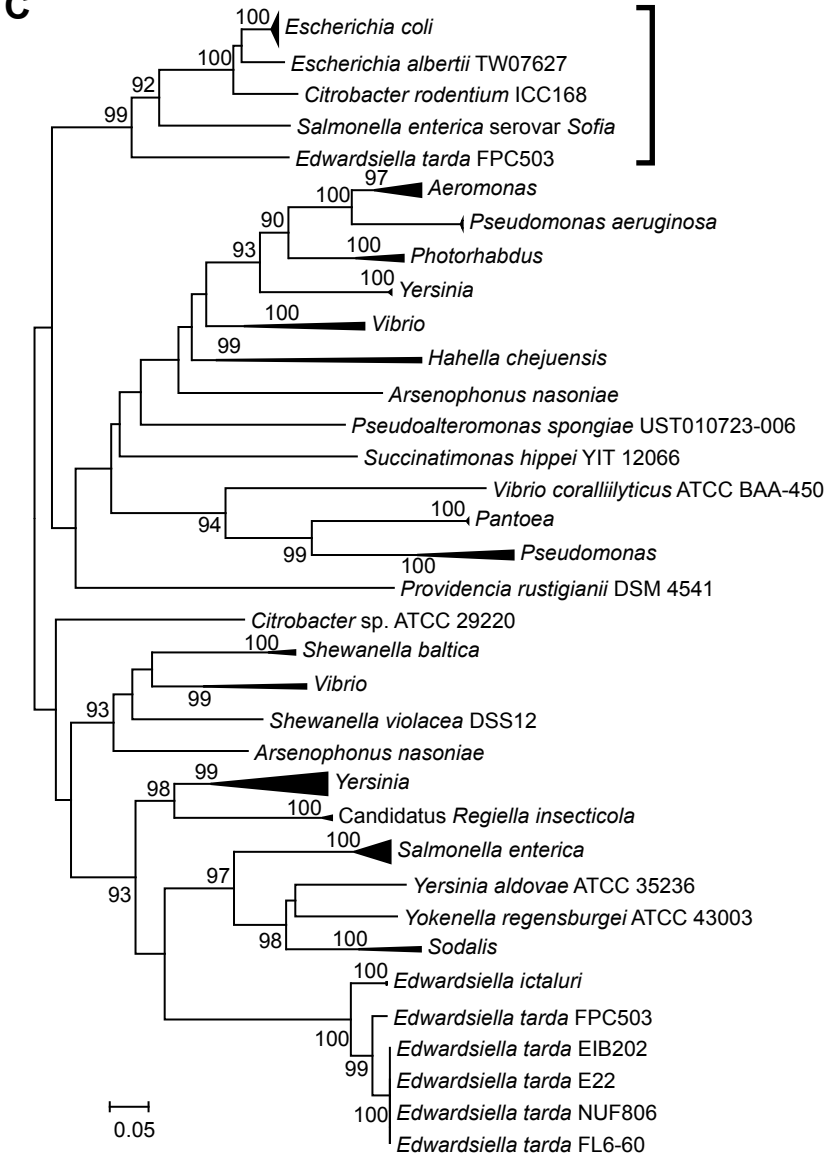

Supplement: Additional file 11: Figure S8 — Phylogenetic trees of T3SS genes. Numbers at the branches indicate the bootstrap probabilities (≥90%) with 1000 replicates. Bracket indicates the clade of LEE genes. (A) Phylogenetic tree based on the escJ gene. (B) Phylogenetic tree based on the escN gene. (C) Phylogenetic tree based on the escR gene. [file 1471-2164-14-642-S11.pdf]
